# Changes in gait performances during walking with head movements in older adults with chronic neck pain

**DOI:** 10.3389/fmed.2024.1324375

**Published:** 2024-02-07

**Authors:** Thanya Madsalae, Tanapat Thongprong, Nithinun Chaikeeree, Rumpa Boonsinsukh

**Affiliations:** Faculty of Physical Therapy, Division of Physical Therapy, Srinakharinwirot University, Nakhonnayok, Thailand

**Keywords:** head motion, gait variability, dual task, fall risk, dynamic balance

## Abstract

**Background:**

Chronic neck pain (CNP) can lead to altered gait which is worse when combined with head movement. Gait parameters for indicating speed and symmetry have not been thoroughly investigated in older adults with CNP. This study aimed to compare gait performance in term of speed and symmetry in older adults with and without CNP during walking with head movement.

**Methods:**

Fifty young older adults, consisting of 36 healthy controls without neck pain (OLDs) and 14 older adults with CNP, participated in the study. Participants completed the Neck Disability Index and Activities-specific Balance Confidence Scale. The 10-Meter Walk Test (10MWT) was used to assess gait performance. Participants were instructed to walk at preferred speed under three different head movement patterns: no head movement (NM), horizontal head movement (HM), and vertical head movement (VM). The Inertial Measurement Unit was used to capture gait performance, and its software was used to analyze gait variables; gait speed, Locomotor Rehabilitation Index (LRI), gait asymmetry index, Phase Coordination Index (PCI).

**Results:**

The CNP group reported moderate neck pain with mild disability in activities of daily living, and less balance confidence than the OLD group (*p* < 0.05). The CNP group showed significantly slower gait speed and lower LRI during walking with both the HM and VM (*p* < 0.05), which corresponded to lower stride length and cadence. The gait asymmetry index in the CNP group was significantly higher than the OLD group during walking with VM (*p* < 0.05), whereas the PCI was significantly higher than the OLD group during walking with both HM and VM (*p* < 0.05).

**Conclusion:**

Chronic neck pain affects both speed and symmetry when walking with head movement. Gait parameters in this study could be implemented to identify changes in speed and symmetry of gait in older adults with CNP who have mild disability and high physical functioning.

## 1 Introduction

One of the most common musculoskeletal conditions in older adults is chronic neck pain (CNP) that can affect static and dynamic postural control and result in numerous of functional deficits, including altered gait (1–5). Cervical afferent inputs play an important role in the cervico-ocular reflex, the cervico-collic reflex, and the cervico-spinal reflex to control postural stability. The complicated connections between sensory afferent inputs from the cervical, visual, and vestibular systems provide head movement and postural control with important information, which is the movement and positioning of the head in space (6).

Alterations in cervical afferent input as a result of chronic neck pain affect the integration of sensorimotor information for postural control, leading to postural instability, as shown in previous studies (3–8). For example, older adults with CNP showed worse standing balance than those without CNP when vision and somatosensory inputs from the feet were reduced (3, 5, 7). The modified Clinical Test of Sensory Integration on Balance (mCTSIB) is frequently used to assess static postural control impairments, which evaluates the effectiveness of sensory integration from the vestibular, visual, and somatosensory systems during various activities requiring balance (3, 4). A previous study in older adults with CNP using the mCTSIB with an altered base of support (mCTSIB-aBoS) evaluated the postural control system (4, 8). Across sensorimotor integration tasks, evidence indicates worse static postural control in older adults with CNP than healthy controls. During standing in the narrow stance (with eyes open on a firm surface) and the comfortable stance (with eyes closed on a firm surface and eyes open on a soft surface), older adults with CNP swayed more than controls in the mediolateral and anteroposterior directions, respectively (4). Poor static postural control during walking is related to a shortened single support phase as well as weight-bearing asymmetry of the lower extremities, which leads to an abnormal gait pattern (9).

Assessment of gait performance, namely, the spatiotemporal variables and variability of gait, is used to investigate changes in dynamic postural control due to aging and movement disorders (10). Gait analysis is frequently used to assess fall risk by researchers and clinicians in the context of health research and clinical practice since gait performance can be easily quantified using portable inertial wearable sensors (11). In addition, gait performance has been determined to be a significant indicator of both functional capacity and physical condition in clinical and home settings (12, 13). The strongest evidence to date indicates that gait speed is a useful indicator of fall risk and should be taken into account as part of a comprehensive assessment of the risk of falling in older adults (14). Preferred walking speed and stride length have been shown to decrease with age (15). Moreover, CNP further decreases gait performance in older adults, as demonstrated by increasing gait cycle duration when performing the 10-Meter Walk Test (10MWT) (4) and worse functional performance in the Timed Up-and-Go test and on the dynamic gait index than healthy controls (5, 7). Additionally, the studies also revealed that older adults with CNP performed worse when their walking was combined with head movement (2, 4).

Most functional daily tasks require 30–50% of the active range of motion of the cervical spine (16). Rotation or horizontal head movement has been observed to be coupled with side bending in the completion of routine activities of daily life, and it is likely more relevant to activities associated with balance (17). Head movements during walking aggravated sudden changes or distinct changes in cervical/vestibular inputs, as demonstrated by significantly longer gait cycle durations, slower self-selected gait speeds, and lower cadence during the 10MWT with horizontal head movement in older adults with CNP (2, 4). Apart from gait speed in general, the Locomotor Rehabilitation Index (LRI), the ratio between self-selected gait speed and optimal gait speed, may be useful to assess gait performance. Conceptually, the LRI determines how close the impaired gait is to the normal gait. It is based on the principles of dynamic similarity and the pendular energy-saving mechanism of walking (metabolic impacts and mechanics of walking), which is not yet established in individuals with CNP (18).

During walking, the cyclic motions of the lower extremities have been considered to be symmetrical naturally (19). One of the key factors influencing gait performance and a predictor of fall risk is gait symmetry (20), which emphasizes the bilateral coordination of swing durations during regular walking (21, 22). Gait asymmetry has been hypothesized to be a greater contributor to the compensatory mechanisms utilized for recovering balance during locomotion than the gait variables themselves; hence, gait symmetry serves as an index of the quality of gait control (23). Previous studies have reported that young adults with CNP walked with a stiffer spine, more asymmetric hip mobility, and a more asymmetric gait than those without CNP during preferred speed walking without head movement (24–26). These findings imply that pain at the neck level may affect the movements of the trunk and lower extremities during walking (27). However, the gait asymmetry index was calculated by comparing only left and right swing timings (gait accuracy) without considering consistency of phase generation. Another important indicator that assesses bilateral coordination of gait and has not been thoroughly investigated in individuals with CNP is the Phase Coordination Index (PCI), which is based on lateral temporal accuracy and consistency (28).

Gait performances, in terms of gait speed and symmetry, often used to predict the fall risk of older adults. There is still lack of information about these gait performances during walking with head movements in older adults with CNP. Findings from this study can increase the understandings regarding the effect of chronic neck pain on gait speed and gait symmetry of older adults. It could also help clinicians select appropriate assessment tool in the clinical setting that can identify dynamic postural impairments in older adults with CNP, facilitating the early implementation of specific interventions designed to reduce the risk of falls.

Therefore, the aim of this study was to compare gait parameters, in term of speed and symmetry during walking with and without head movements, of older adults with chronic neck pain (the CNP group) with those without chronic neck pain (the OLD group). Head movement in the vertical and horizontal planes was used in this study to trigger sudden changes in cervico-vestibular inputs, which led to a perturbation of dynamic postural during walking. We hypothesized that both speed and symmetry during walking would be worse in the CNP group compared to the OLD group, and that the difference would be more apparent during head movement.

## 2 Materials and methods

### 2.1 Participants

The sample size was estimated using data from a previous study (26). A sample size of at least 12 individuals in each group is needed to provide a sufficient power of 0.80 for the Mann–Whitney U test when the alpha level is set at 0.05, as performed by power analysis with G*Power version 3.1.9.4. However, this study included a larger sample size in the control group for an accurate comparison. Thus, a convenience sampling technique was used to recruit 14 older adults with CNP (CNP = 14) and 36 controls (OLDs = 36). All participants aged 60 years or older were capable of walking independently and without the use of walking aids. Neck stiffness and pain, either alone or in combination with radiating neck pain, were considered to be symptoms of neck pain. Participants in the CNP group were required to have reported neck pain as the primary complaint for at least 3 months, and their average weekly pain severity needed to be at least 3 cm out of 10 cm on the Visual Analogue Scale (VAS) in order to be eligible (29).

Participants were not allowed to participate if they had comorbidities that could have an impact on balance measurements, including: recent fracture or orthopedic surgery (within the past 6 months), a history of neck and head trauma, recent inflammatory joint disease/arthritis or acute musculoskeletal injury requiring active management, systemic diseases, systemic conditions, neurological diseases, dizziness or vertigo caused by ear or brain disorders, suspected or diagnosed vestibular disease, the usage of medications that affected balance, or cognitive impairment with a score below 24 out of 30 on the Montreal Cognitive Assessment (MoCA) (30, 31).

The participants were recruited through public advertisements. The pre-screening was completed via mobile phone to acquire information in order to determine if they were a suitable candidate for further processing. Subsequently, the eligible participants were scheduled to come in person for further assessment of postural control.

Ethics approval for the study was granted by the Human Research Ethics Committee of Srinakharinwirot University (SWUEC-039/2562F). Written informed consent was obtained before participation.

### 2.2 Procedures

This cross-sectional study was designed as a single-blind study, with only one rater (Rater 2) responsible for conducting all postural control assessments and being blinded to the demographic data and participant’s group. After obtaining informed consent, Rater 1 collected demographic data. All postural control assessments were conducted in a quiet laboratory setting by Rater 2. Rater 2’s intrarater reliability on the mCTSIB-aBoS was calculated using the intraclass correlation coefficient (ICC) for a sample of 10 older adults. The findings demonstrated that Rater 2 had a high intrarater reliability (ICC = 0.98). The postural control assessments were organized into 11 sequences that began with different tasks of each assessment and continued with the subsequent tasks. In the first sequence, eyes open while standing comfortably on a firm surface (C1) of the mCTSIB-aBoS was assigned first, followed by eyes open while standing narrowly on a firm surface (C2), eyes closed while standing comfortably on a firm surface (C3), eyes closed while standing narrowly on a firm surface (C4), eyes open while standing comfortably on a soft surface (C5), eyes open while standing narrowly on a soft surface (C6), eyes closed while standing comfortably on a soft surface (C7), and eyes closed while standing narrowly on a soft surface (C8), walk at their preferred speed barefoot with no head movement (NM), horizontal head movement or turning the head left and right as far as was comfortable (HM), and vertical head movement or turning the head up and down as far as was comfortable (VM), while in the second sequence, C2 of the mCTSIB-aBoS was assigned first, followed by C3, C4, C5, C6, C7, C8, NM, HM, VM, and C1. All participants were randomly assigned to one of the 11 sequences, and the researcher made sure that each sequence had an equal number of participants aimed to prevent order bias influenced by respondent fatigue and item difficulty (32). Each participant was given time to familiarize themselves with the testing procedures and instructions prior to data collection and completed all postural control assessments once.

Participants were advised to take a 5-min break in between trials to prevent fatigue. The entire testing session took approximately 40 min. For subsequent review, each participant was videotaped during testing.

### 2.3 Measurement tools

Numerous clinical assessments were used. The demographic characteristics were gathered via inquiries from patients and clinical records. Participants completed questionnaires assessing age, gender, lower limb length, body mass index (BMI), comorbidities, medication usage, neck pain and disability, handicap related to dizziness, and confidence in balancing.

#### 2.3.1 Questionnaires

The severity of neck pain was assessed using a Visual Analogue Scale (VAS) with a 0–10 rating, where 0 indicated no pain and 10 indicated the worst imaginable pain. To evaluate the level of self-reported neck pain and disabilities, the Thai version of the Neck Disabilty Index (NDI) (33) was administered using an interviewer-assisted questionnaire. There are a total of 10 items included with the subjects of daily tasks, pain, and concentration. With a maximum possible score of 50 (or 100%), each item is given a score between 0 and 5, with 0 indicating no disability and 5 indicating severe disability. The overall scores are then converted into one of five levels of disability in terms of activities of daily living: no disability (0–8%), mild disability (10–28%), moderate disability (30–48%), severe disability (50–64%), and complete disability (70–100%) (34).

The Thai version of the Activities-specific Balance Confidence scale (ABC) was used to assess level of confidence in maintaining balance while completed 16 different activities on an 11-point scale (from 0 to 100%). Each item outlines a unique challenge activity that requires an increasing degree of balance control. Higher scores indicate greater balance confidence (35).

The self-perceived handicap related to dizziness was evaluated using the Dizziness Handicap Index (DHI). Functional disability, emotional disability, and physical disability are the three subscales of DHI, which consists of 25 items. With a maximum score of 100, higher values imply more perceived impairment. The DHI categorizes people into three degrees of disability: mild disability (0–30), moderate disability (31–60), and severe disability (61–100) (36).

#### 2.3.2 Clinical balance tool

The Modified Clinical Test of Sensory Integration on Balance with Alternate Base of Support (mCTSIB-aBoS), which was developed to systematically assess the impact of visual, vestibular, and somatosensory input on standing balance (37), is a timed test that has been shown to have good test–retest reliability in older populations (38). Participants, similar to those in the previous study of older adults with CNP (8), were asked to stand with their arms crossed for 30 s as stably as possible under eight different conditions: C1-C8. When participants opened their eyes, moved an arm or both, or took a step, the trial ended. The time taken to finish the trial was recorded. The total score was calculated by averaging the times on all conditions.

The Ten-Meter Walk (10MWT) test, in which participants select a preferred walking speed over a distance of 10 meters, is widely used and recommended as a physical mobility and balance test (39). The 10MWT has been hypothesized to be a primary predictor of self-perceived function (40). The participants were given instructions to walk at their preferred speed barefoot under three different movements of the head: (1) NM; (2) HM; and (3) VM.

#### 2.3.3 Inertial measurement units

Gait parameters, including gait speed, stride length, and cadence during the walking test, were collected with the Instrumented Long Walk (IWalk) test (APDM, Inc., Portland, United States). Six inertial measurement units (IMUs, Inc., Portland, OR, USA) were worn by all participants at the chest, lumbar region, wrists, and shanks (4 cm above the ankle joints) before performing the clinical test, with each sensor consisting of a gyroscope and an accelerometer to record angular velocity and linear acceleration at a sampling rate of 200 Hz (41). The instrument has high to moderate validity when measuring most of the gait metrics tested (42). The IMUs at both shanks are used to detect and evaluate self-selected walking speed (SSWS), stride length, and cadence.

The optimal walking speed (OWS) which is the speed that the metabolic cost of walking is lowest was performed using an equation proposed by a previous study (43). In this equation, the Froude Number is denoted as 0.25, the gravitational acceleration is represented by 9.81 m/s^2^, and the lower limb length (LLL) of each participant was considered from the distance between the floor to the greater trochanter of the femur through the lateral malleolus.

$$OWS=\sqrt{0.25*9.81*LLL}$$

The Locomotor Rehabilitation Index (LRI), expressed as a percentage value, was calculated using the equation below (43). Lower LRI values indicate a significant metabolic expense (cost of transport) due to an impaired pendular mechanism.

LRI=100×SSWS/OWS

To allow comparison to normative data, the value was averaged for the left and right sides in relation to the subject’s body height (44). The data in percentage of gait cycle’s swing phase from the left and right sides were compared to determine the short and long swing phase. The term “short swing phase” (SSW) refers to the value that is smaller than that of the “long swing phase” (LSW).

Gait asymmetry was calculated using the proportion of a gait cycle’s swing phase when each foot was off the ground. The gait asymmetry was calculated by multiplying 100 by the absolute value of the natural logarithm of the number obtained by dividing the SSW by the LSW. When the value increases, it reflects the degree of gait asymmetry. A value of zero denotes complete symmetry (26).

GaitAsymmetryIndex=100×lnSSWLSW

The Phase Coordination Index (PCI) (28) was used to identify the degree of accuracy and consistency of left–right stepping. The stride time of each foot was computed from the time required to complete one gait cycle or 360° of a cyclical movement. The phase $\varphi_{i}$ was defined as the relative timing of contralateral heel strikes, which would ideally be 180° for each step.

$$\varphi_{i}=360{}^{\circ}\times \;\frac{\mathrm{Step}\;Time_{i}}{\mathrm{Stride}\;Time_{i}}$$

Gait Accuracy is percentage of mean absolute differences of all φ from the standard degree of 180, and Gait Consistency is the percentage of the coefficient of variation of Gait Accuracy.

$$\begin{array}{c} \mathrm{Gait}\;\mathrm{Accuracy}\;\left(\%\right)=\frac{\bar{|\varphi_{i}-180{}^{\circ}|\;}}{180{}^{\circ}}\times \;100;\\ \mathrm{Gait}\;\mathrm{Consistency}\;\left(\%\right)=\frac{SD_{\mathrm{Gait}\;\mathrm{Accuracy}}}{Mean_{\mathrm{Gait}\;\mathrm{Accuracy}}} \end{array}$$

The bilateral coordination of gait or PCI was computed by combining the Gait Accuracy and Consistency values.

$$PCI\;\left(\%\right)=\mathrm{Gait}\;\mathrm{Accuracy}+\mathrm{Gait}\;\mathrm{Consistency}$$

Lower PCI values indicate greater accuracy and consistency of bilateral gait coordination, whereas higher PCI values indicate less accuracy and consistency (28).

### 2.4 Statistical analysis

To provide an overview of the demographic data, descriptive statistics were used. The normality of the data was determined using the Shapiro–Wilk goodness-of-fit test, which showed no evidence of being normally distributed. As a result, the Mann–Whitney U test was selected to compare the total score of the mCTSIB-aBoS, gait speed, stride length, cadence, PCI, LRI, and gait asymmetry index between older adults with and without CNP (reported using U-value). The Kruskal–Wallis test was chosen to investigate the differences in outcome variables among the three different head movement patterns within the group: walking without head movement, walking with horizontal head movement, and walking with vertical head movement (reported using H-value). All analyses were conducted with a significance level of 0.05. The effect size of 0.2 to 0.5 were considered small, 0.5 to 0.8 were considered medium, and greater than 0.8 were considered large (45). A greater effect size indicates that research finding has greater practical significance, whereas a smaller effect size indicates restricted practical applicability.

## 3 Results

The demographic data of older adults without chronic neck pain (OLDs) and older adults with chronic neck pain (CNP) are presented in Table 1. No significant differences in gender, age or comorbidities between older adults with and without CNP were found. Most of the participants in both groups were female, without significant difference in body mass index between groups. According to the NDI score, the CNP group reported none to mild disability in activities of daily living caused by neck problems, which was significantly greater than that experienced by the OLD group (*p* < 0.001). The pain level of the CNP group was classified as moderate intensity. Based on the ABC scale, those with CNP had less balance confidence than the OLDs (*p* = 0.021). Additionally, the total scores on the mCTSIB-aBoS, which represent static postural control, were significantly lower in the CNP group (*p* < 0.001).

**Table 1:** Characteristics of participants.

	OLD (*n* = 36)		CNP (*n* = 14)	
	Median	95% CI	Median	95% CI
Age (years)	64.00	(62.00 to 65.00)	63.00	(61.00 to 66.00)
BMI (kg/m^2^)	22.86	(22.66 to 23.34)	22.55	(20.89 to 23.53)
NDI (0–100)	0.00	(0.00 to 0.00)	14.00	(8.00 to 20.00)
ABC scale (0–100)	95.63	(95.00 to 98.63)	93.13	(88.13 to 96.81)*
DHI (0–100)	0.00	(0.00 to 0.00)	0.00	(0.00 to 0.00)
VAS (0–10)	N/A	N/A	45.00	(30.00 to 60.00)*
Duration of neck pain (months)	N/A	N/A	18.00	(3.50 to 24.00)*
mCTSIB-aBoS (% total score)	100.00	(100.00 to 100.00)	100.00	(98.75 to 100.00)*
Gender [female, *n* (%)]	29 (80.56)	–	11 (78.57)	–
Side of neck pain [sides, *n* (%)]				
Right side	N/A	–	3 (21.43)	–
Left side	N/A	–	2 (14.29)	–
Both side	N/A	–	9 (64.29)	–

95% CI = 95% confidence interval; NDI, neck disability index; ABC scale, activities-specific balance confidence scale; DHI, dizziness handicap index; VAS, visual analog scale; mCTSIB-aBoS, modified clinical test of sensory integration on balance; OLD, older adults without chronic neck pain; CNP, older adults with chronic neck pain, N/A, not applicable.

*p < 0.05 comparison of OLD and CNP.

**Figure 1:**
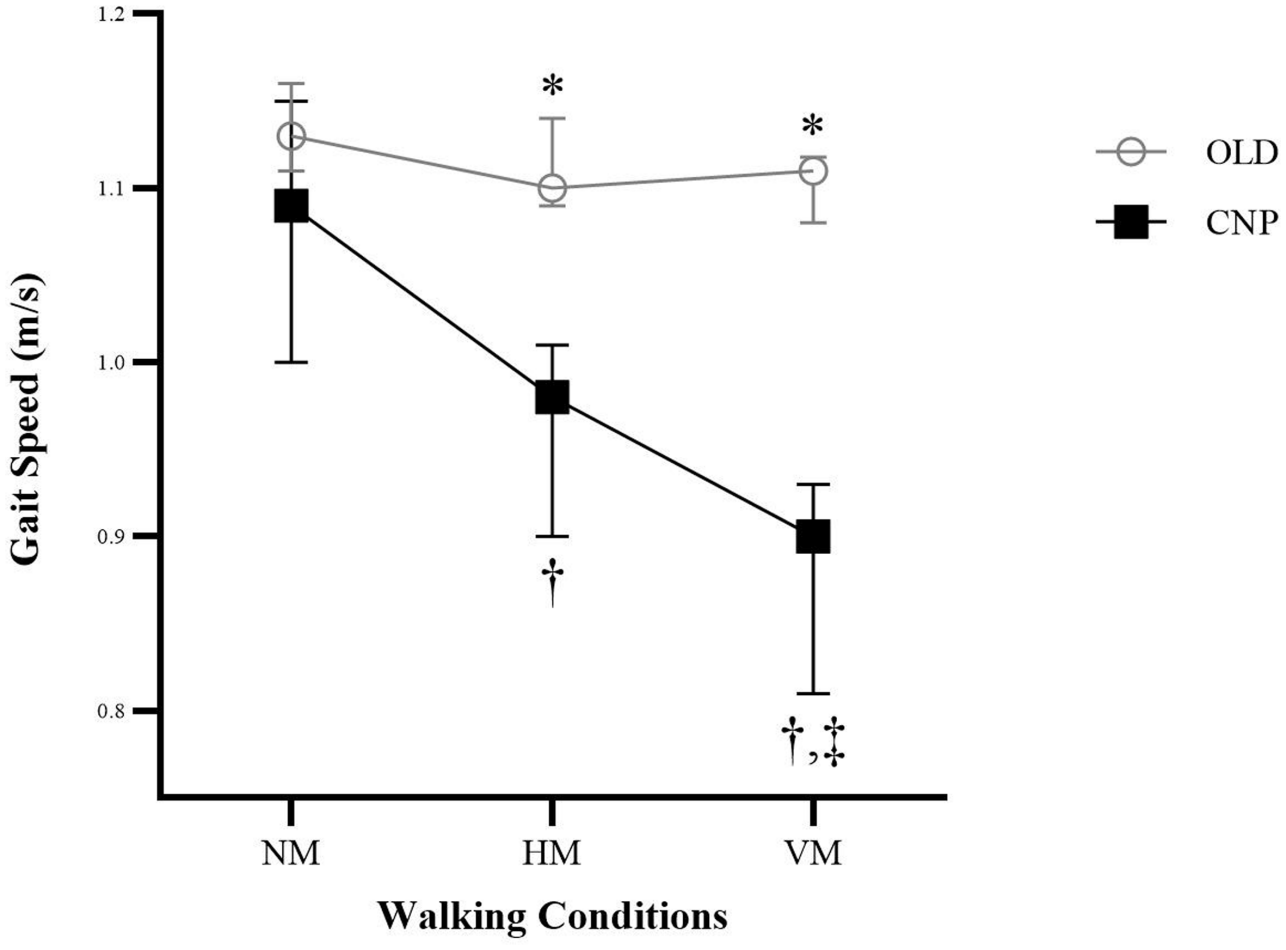
Gait Speed during walking with three different head movement patterns between the Control (OLD) and Neck Pain Groups (CNP); There was no difference across the head movement patterns during the 10-Meter Walk Test in the OLD group. *Significant difference in gait speed between group were found during walking with Horizontal Head Movement (HM) and Vertical Head Movement (VM); †The pairwise comparison within the CNP group showed significant difference in gait speed between walking with No Head Movement (NM) and HM, and between NM and VM; ‡The pairwise comparison within the CNP group showed significant difference in gait speed between HM and VM.

The spatiotemporal variables are presented in Table 2. There were no differences in gait speed, stride length, or cadence between the OLD and CNP groups during walking with no head movement (NM). However, the CNP group had significant lower gait speed, stride length, and cadence (*p* < 0.001) than the OLD group during walking with horizontal head movement (HM) and vertical head movement (VM). Furthermore, gait speed was significantly different among the three different head movement patterns of 10MWT in the CNP group (*H*-value = 24.83, effect size = 0.59; *p* < 0.001); it was highest during walking with NM and lowest during walking with VM, as shown in Figure 1. Similar to gait speed, the Locomotor Rehabilitation Indices (LRIs) in Table 3 show significant differences between the OLD and CNP groups during walking with HM and VM (*p* < 0.001) and also significantly different among the three different head movement patterns of 10MWT in the CNP group (H-value = 27.02, effect size = 0.64; *p* < 0.001). The LRIs during walking with HM and VM were 74.57 and 68.14%, respectively, both of which were lower than walking with NM (82.86%). This indicates that walking with head movement resulted in decreased gait speed compared to the optimal gait speed.

**Table 2:** Gait Parameters during walking with three different head movement patterns between the control and neck pain groups.

Gait parameter	Head movement patterns of 10MWT	OLD (*n* = 36)		CNP (*n* = 14)		Value of *p*	*U-*value	Effect size
		Median	95% CI	Median	95% CI			
Gait speed (m/s)	Walk without head movement	1.13	(1.11 to 1.16)	1.09	(1.00 to 1.15)	0.054	163.00	0.27
	Walk with horizontal head movement	1.10	(1.09 to 1.14)	0.98	(0.90 to 1.01)	<0.001*	36.50	0.66
	Walk with vertical head movement	1.11	(1.08 to 1.11)	0.90	(0.81 to 0.93)	<0.001*	1.00	0.77
Stride length (cm.)	Walk without head movement	124.03	(120.59 to 127.73)	120.42	(115.99 to 123.23)	0.060	165.00	0.27
	Walk with horizontal head movement	117.42	(114.07 to 121.58)	109.00	(104.81 to 111.55)	<0.001*	96.00	0.48
	Walk with vertical head movement	117.00	(115.27 to 121.91)	110.23	(103.98 to 113.10)	0.004*	117.00	0.41
Cadence (steps/min)	Walk without head movement	107.43	(104.72 to 109.47)	103.41	(99.55 to 108.62)	0.101	176.00	0.23
	Walk with horizontal head movement	102.60	(100.84 to 104.59)	96.82	(91.74 to 100.90)	0.002*	112.00	0.43
	Walk with vertical head movement	105.58	(102.69 to 107.24)	94.34	(92.64 to 101.02)	<0.001*	68.00	0.56

10MWT = The 10-meter walk test; OLD, older adults without chronic neck pain; CNP, older adults with chronic neck pain.

95% CI = 95% confidence interval.

*p < 0.05 comparison of OLD and CNP.

**Table 3:** Locomotor Rehabilitation Index during walking with three different head movement patterns between the control and neck pain groups.

Head movement patterns of 10MWT	OLD (*n* = 36)		CNP (*n* = 14)		Value of *p*	*U-*value	Effect size
	Median	95% CI	Median	95% CI			
Walk without head movement (%)	85.87	(83.22 to 88.33)	82.86	(77.91 to 87.18)	0.101	176.00	0.23
Walk with horizontal head movement (%)	83.96	(81.93 to 86.65)	74.57	(70.14 to 76.06)	<0.001*	24.00	0.70
Walk with vertical head movement (%)	83.42	(82.32 to 84.71)	68.14	(62.79 to 71.74)	<0.001*	0.00	0.77

10MWT = The 10-meter walk test; OLD, older adults without chronic neck pain; CNP, older adults with chronic neck pain.

95% CI = 95% confidence interval.

*p < 0.05 comparison of OLD and CNP.

**Figure 2:**
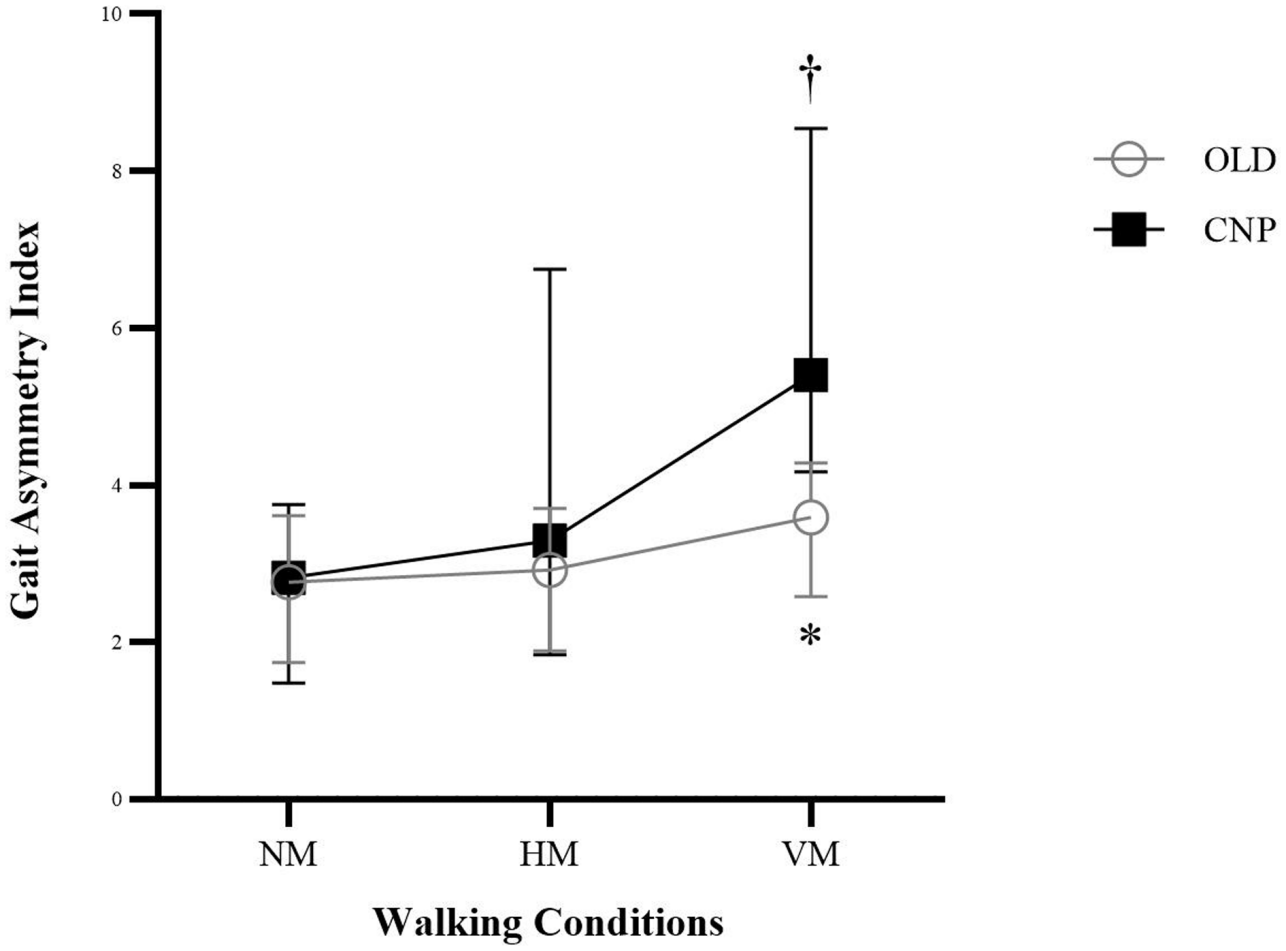
Gait Asymmetry Index during walking with three different head movement patterns between the Control (OLD) and Neck Pain Groups (CNP); There was no difference across the head movement patterns during the 10-Meter Walk Test in the OLD group. *Significant difference in gait asymmetry index between group were found during walking with Vertical Head Movement (VM); †The pairwise comparison within the CNP group showed significant difference in gait asymmetry index between walking with No Head Movement (NM) and VM, while no significant difference was found when comparing both NM and VM to walking with Horizontal Head Movement (HM).

Gait asymmetry indices under the three different head movement patterns of 10MWT in the OLD and CNP groups are presented in Table 4. Compared to the OLD group, the CNP group had the largest gait asymmetry index when walking with VM (*p* = 0.006; Table 4). Further investigation within groups revealed a significant difference in the gait asymmetry index between walking with NM and walking with VM in the CNP group (H-value =12.79, effect size = 0.45, *p* = < 0.001), as shown in Figure 2. Whereas Table 5 shows that the Phase Coordination Indices (PCIs) in the CNP group were significantly higher than the OLD group during walking with HM and VM (*p* = 0.015 and *p* < 0.001, respectively). Also, within the CNP group, the PCIs were significantly different in all head movement patterns of 10MWT (H-value = 31.62, effect size = 0.76; *p* < 0.001), which get worst during walking with VM (8.68%).

**Table 4:** Gait Asymmetry Index during walking with three different head movement patterns between the control and neck pain groups.

Head movement patterns of 10MWT	OLD (*n* = 36)		CNP (*n* = 14)		Value of *p*	*U-*value	Effect size
	Median	95% CI	Median	95% CI			
Walk without head movement	2.76	(1.74 to 3.61)	2.82	(1.48 to 3.75)	0.730	236.00	0.05
Walk with horizontal head movement	2.92	(1.89 to 3.70)	3.29	(1.84 to 6.75)	0.342	208.00	0.13
Walk with vertical head movement	3.59	(2.58 to 4.28)	5.40	(4.17 to 8.54)	0.006*	125.00	0.39

10MWT = The 10-meter walk test; OLD, older adults without chronic neck pain; CNP, older adults with chronic neck pain.

95% CI = 95% confidence interval.

*p < 0.05 comparison of OLD and CNP.

**Table 5:** Phase Coordination Index during walking with three different head movement patterns between the control and neck pain groups.

Head movement patterns of 10MWT	OLD (*n* = 36)		CNP (*n* = 14)		Value of *p*	*U-*value	Effect size
	Median	95% CI	Median	95% CI			
Walk without head movement (%)	5.00	(4.52 to 5.94)	5.53	(5.09 to 6.23)	0.503	283.00	0.09
Walk with horizontal head movement (%)	6.28	(6.20 to 6.74)	7.11	(6.42 to 7.60)	0.015*	365.00	0.35
Walk with vertical head movement (%)	7.43	(7.03 to 7.93)	8.68	(8.55 to 9.78)	<0.001*	450.00	0.61

10MWT = The 10-Meter Walk Test; OLD = Older Adults without Chronic Neck Pain; CNP = Older Adults with Chronic Neck Pain.

95% CI = 95% confidence interval.

*p < 0.05 comparison of OLD and CNP.

## 4 Discussion

This study aimed to compare gait performance in term of speed and symmetry, during walking with head movement in older adults with and without chronic neck pain. The CNP group exhibited slower gait speed with shorter step length and decreased cadence, as well as a lower Locomotor Rehabilitation Index (LRI) and a higher Phase Coordination Index (PCI) under walking with horizontal head movement (HM) and vertical head movement (VM) compared to older adults without chronic neck pain (the OLD group). However, the gait asymmetry index significantly differed between groups only when walking with VM.

The findings of slower gait speed and other altered gait parameters such as stride length and cadence in older adults with CNP during walking with head movements were consistent with the findings of previous studies (46). Self-selected or preferred gait speed is a predictor of self-perceived function (40). Previous studies demonstrated that older adults with a history of falls had shorter stride length and slower gait speed than the control group (47–49). Alterations in gait performance seen in current study could indicate a higher risk of falls in those with CNP compared to the OLDs. Decreased lower extremity muscle strength may explain the altered gait parameters in older adults with CNP. Previous study found that older adults with CNP demonstrated significantly reduced hip/trunk lateral strength in the comprehensive balance test (Balance Evaluation Systems Test; BESTest) (1). Additionally, multiple gait parameters are correlated with the strength of multiple lower extremity muscles in older adults, particularly women (50).

Significant alterations in gait speed, LRI, and PCI were seen in the CNP group when walking with HM and VM in the current study. Sustained abnormal afferent input could disturb sensory system integration and lead to subsequent impairment of the vestibular system. Furthermore, abnormalities in the cervical spine, either ischemia of the vertebral arteries or a malfunction in the neck’s proprioceptive system, could affect vestibular nuclei (51). Abnormal afferent inputs from the somatosensory and/or vestibular systems at the neck level might lead to greater gait disturbances while walking with head movement (6). Stabilizing the trunk in space and facilitating intersegmental movements is the primary function of vestibulospinal control. Changes in vestibular signals could lead to higher trunk variability and disrupt the trunk-leg phase (52). In addition, visual, vestibular, proprioceptive, and somatosensory input all play a key role in walking ability (53). Abnormal afferent inputs from the cervical spine have been proposed to affect the signal integration of the sensorimotor control system (6, 54). According to numerous studies, neck pain can affect proprioceptive function, posture, oculomotor control, and hand-eye coordination (6, 55, 56). Cervical spine functional and structural abnormalities may change proprioceptive functions, joint mechanics, and muscle spindle sensitivity, resulting in postural instability and reduced gait speed (6, 54). Thus, the abnormal gait performance in older adults with CNP may be caused by the malfunction of one or more sensorimotor control system components, as partly observed in the lower total scores of the mCTSIB-aBoS in the CNP group.

The CNP group exhibited poor bilateral coordination of phase when walking with HM and VM compared to walking with no head movement (NM) and compared to the OLD group. The incoordination of gait pattern may be a compensatory strategy for the instability demonstrated by those with CNP when stability is challenged. It has been hypothesized that abnormal cervical afferent inputs could lead to the asymmetric gait seen in older adults with CNP. In general, it is observed that neck pain often develops unilaterally or exhibits more severity on one side than the other (57) This asymmetry in pain might lead to asymmetrical sensory inputs, consequently having a significant impact on postural control, orientation, and the perception of body schema (58–60). According to a recent study, it has been proposed that individuals experiencing CNP may exhibit a distorted body schema (61). The integration of sensory feedback originating from the lower extremities plays an important part in the establishment of neuronal loops between central pattern generators located in the spinal cord during the process of walking (21). The distorted body schema caused by neck pain can lead to modulation of sensory feedback, potentially resulting in altered motor responses in both lower extremities. This can lead to impaired bilateral coordination of phase while walking with head movement in individuals with CNP. In contrast, no asymmetrical gait pattern was observed in the CNP group during walking with HM. A possible explanation could be that bilateral gait compensation occurred due to turning the head right and left; thus, an asymmetrical pattern was not observed. Most of the head movements associated with balance control in daily activities occur when the head is moved horizontally, rather than vertically, since this kind of movement is more relevant to typical daily tasks (17). Moreover, the normal field of the vision is typically 180° horizontally (160° for monocular vision) and 135° vertically (62); thus, a person may better compensate for HM than for VM. Indeed, asymmetrical gait differed during walking with VM compared to during HM. These explanations could be the reasons why all gait parameters were found to be the worst when walking with VM.

Walking with head movement can be classified as performing a dual motor task. The findings may also imply that it is difficult for older adults with CNP to perform two tasks simultaneously. Lots of evidence has shown that gait disturbances during a complex gait task have been associated with CNP (2, 4, 26, 56, 63). Dual task performance evaluation could be used to differentiate fallers from non-fallers. There were no variations in gait parameters between fallers and non-fallers in a single-task condition; however, there were significant differences while performing an additional task while walking (64). These findings are in agreement with our findings that a challenging condition, such as head movement while walking, was required to observe the gait disturbances caused by the alteration of afferent inputs from the cervical region in older adults with CNP.

Results of this study suggest that older adults with CNP have poorer gait control than those without CNP during walking with head movement, especially in the vertical plane. Gait speed and symmetry are also useful measures for identifying dynamic postural control impairments in older adults with CNP, as evidenced by slower gait speed, LRI and PCI during waking with both HM and VM. Measurement of gait speed during walking with head movement can be further used to evaluate the risk of falls in clinical or community settings. Furthermore, older adults with CNP were found to have poor bilateral coordination of gait when walking with head movement compared to walking without head movement. Specific intervention designed to target bilateral coordination of gait may potentially improve functional mobility and balance control in older adults with CNP. Nevertheless, the current study was unable to clarify whether gait disturbances are caused by sustained abnormal afferent inputs from cervical or an additional vestibular disturbance. Therefore, additional research is required in order to better understand the mechanisms that contribute to gait disturbances during walking with head movement in individuals with CNP.

The study’s findings should be interpreted in light of its limitations. To some extent, this study controlled the magnitude of head movement during walking, but these magnitudes were still different in individuals with CNP, depending on the available range of head movement and pain. The readers should be aware that self-selected head movement could lead to the variability of perturbation amplitude which may influence sensory systems differently among individuals with CNP. Furthermore, the majority of the CNP group experienced moderate pain with no or mild disability during activities of daily living. The severity of the condition may differ across patients with varying degrees of pain and disability. The older adults in the CNP group with any type of pathology associated with balance control were excluded to control for the effects of aging, resulting in a small sample size. It is widely known that such adults have many health problems and complications in general; consequently, a greater severity of dynamic postural control impairments is expected in a broader group of older adults with CNP. Nonetheless, older adults with CNP and other comorbidities should be recruited in future studies to confirm this hypothesis.

## 5 Conclusion

Older adults with chronic neck pain (CNP) have gait disturbances, in term of speed and symmetry, during walking with head movements, as compared to older adults without neck pain. Gait disturbances in CNP may be a compensatory strategy for gait instability caused by abnormal afferent inputs from the cervical spine.

## Data availability statement

The raw data supporting the conclusions of this article will be made available by the authors, without undue reservation.

## Ethics statement

The studies involving humans were approved by the Human Research Ethics Committee of Srinakharinwirot University (SWUEC-039/2562F). The studies were conducted in accordance with the local legislation and institutional requirements. The participants provided their written informed consent to participate in this study.

## Author contributions

TM: Conceptualization, Data curation, Formal analysis, Investigation, Validation, Writing – original draft, Writing – review & editing. TT: Data curation, Investigation, Writing – original draft. NC: Methodology, Resources, Software, Visualization, Writing – review & editing. RB: Conceptualization, Data curation, Formal analysis, Funding acquisition, Methodology, Project administration, Resources, Supervision, Validation, Writing – original draft, Writing – review & editing.
